# A biomedical event extraction method based on fine-grained and attention mechanism

**DOI:** 10.1186/s12859-022-04854-0

**Published:** 2022-07-29

**Authors:** Xinyu He, Ping Tai, Hongbin Lu, Xin Huang, Yonggong Ren

**Affiliations:** 1 School of Computer and Information Technology, Liaoning Normal University, Dalian, Liaoning China; 2 Information and Communication Engineering Postdoctoral Research Station, Dalian University of Technology, Dalian, Liaoning China; 3 Postdoctoral Workstation of Dalian Yongjia Electronic Technology Co., Ltd, Dalian, Liaoning China; 4 FIL Technology Limited, Dalian, Liaoning China; 5 Anshan Normal University, Anshan, Liaoning China

**Keywords:** Event extraction, Fine-grained, Trigger identification, Argument detection, Multi-level attention

## Abstract

**Background:**

Biomedical event extraction is a fundamental task in biomedical text mining, which provides inspiration for medicine research and disease prevention. Biomedical events include simple events and complex events. Existing biomedical event extraction methods usually deal with simple events and complex events uniformly, and the performance of complex event extraction is relatively low.

**Results:**

In this paper, we propose a fine-grained Bidirectional Long Short Term Memory method for biomedical event extraction, which designs different argument detection models for simple and complex events respectively. In addition, multi-level attention is designed to improve the performance of complex event extraction, and sentence embeddings are integrated to obtain sentence level information which can resolve the ambiguities for some types of events. Our method achieves state-of-the-art performance on the commonly used dataset Multi-Level Event Extraction.

**Conclusions:**

The sentence embeddings enrich the global sentence-level information. The fine-grained argument detection model improves the performance of complex biomedical event extraction. Furthermore, the multi-level attention mechanism enhances the interactions among relevant arguments. The experimental results demonstrate the effectiveness of the proposed method for biomedical event extraction.

## Background

With the rapid development in the fields of Natural Language Processing (NLP) and Text Mining (TM), the study on event extraction has gained great popularity. Event extraction is an effective way to represent the structured knowledge from unstructured text [1]. Biomedical event extraction, as the pivotal task of biomedical text mining, is helpful to provide enlightenment and basis for drug research and disease diagnosis. Also, there are many useful applications for biomedical event task, such as domain search engine [2], pathway curtain [3] and so on. Meanwhile, many evaluation tasks have been organized for providing novel methods of biomedical event extraction tasks, such as BioNLP 2009 [4], BioNLP 2011 [5], BioNLP 2013 [6], and BioNLP 2016 [7].

According to the BioNLP [4], a biomedical event consists of an event trigger word and a set of arguments. Event trigger is usually a verb or gerund phrase that describe the occurrence of a biomedical event. Each event trigger has a specific type, which represents the event type. Arguments denote the participants of biomedical events, which are generally represented as relation pairs of event triggers and entities or triggers and other events. Therefore, biomedical event extraction aims to identify the event triggers and detect their arguments from the biomedical literature, then construct complete biomedical events. Biomedical events can be divided into simple events and complex events. The simple events usually include one trigger and one argument. The complex events consist of multiple arguments, and there may be nested events, that is, the event arguments are other events. Due to the complexity of complex biomedical event structure, the performance of complex event extraction is relatively low.

**Fig. 1:**
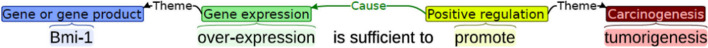
A sentence with visualized events provided by BionNLP-ST2013

Figure 1 gives an example provided by BionNLP-ST2013. In the sentence “Bmi-1 over-expression is sufficient to promote tumorigenesis” of Fig. 1, there exists a *Gene expression* type simple event with a trigger word “over-expression”, and a *Theme* type argument “Bmi-1” which is an entity. In addition, there exists a complex *Positive regulation* type event, that is event nested with other events with a trigger word “promote”. This event has a *Theme* type argument “tumorigenesis” and a *Cause* type argument linked to *Gene expression* event “over-expression”.

Many advanced methods have been proposed for biomedical event extraction. The previous work can be divided into three categories: rule-base methods, traditional machine learning approaches and deep learning models. The rule-based methods [8, 9] focus the formulation of extraction rules and the generation of pre-defined dictionary, which are time-consuming and difficult to cover all types. Machine learning methods are currently the common approaches for biomedical event extraction. For the MLEE dataset, Pyysalo et al. [10] utilized a SVM classifier for biomedical event extraction, integrating context and dependency features. Zhou et al. [11] proposed a semi-supervised learning model to extract biomedical events by un-annotated corpus and hidden topics. In addition, some researchers pay more attention on the biomedical event trigger identification, which is the sub-task of biomedical event extraction. Zhou et al. [12] obtained biomedical domain knowledge and embedded it into word features, then they combined the embedded features and context features for trigger identification. Our previous work [13] have proposed a two-stage biomedical event trigger detection method, which employed SVM and PA algorithm for classification integrating rich manual features and feature selection. For biomedical event extraction, pipeline-based systems are popular and the feasibility of these methods are verified on many datasets, such as TEES [14, 15], EventMine [16]. The aforementioned methods rely on the handcrafted features, and tailor different features for specific task which may require excessive experiments.

In recent years, various neural networks have been applied into biomedical event extraction task successfully. Wang et al. [17] have proposed a CNN architecture for biomedical event extraction. They integrated multiple distributed representation, such as trigger types, POS labels and topic representation. Li et al. [18] employed GRU neural network to extract biotope and bacteria events which focus on detecting the relationship between two mandatory argument, the bacterium and location. They integrated attention mechanism to enhance the important information and employed a domain-oriented word representation. Yan et al. [19] built a bottom-up detection framework based on LSTM to identify the biotope and bacteria events. They trained the context embedding model (VecEntNet) using the annotations of arguments. The context embeddings are further adopted to train the event detection model (VecComNet) for detecting event type and direction. However, the deep learning model adopted in VeComNet is limited by the number of training samples. Abdulkadhar et al. [20] presented a hybrid approach that integrates an ensemble-learning framework by combining a Multiscale Laplacian Graph kernel and a feature-based linear kernel, using a pattern-matching engine to identify biotope and bacteria events. In addition, for the biomedical event trigger detection, Nie et al. [21] proposed a word embeddings assisted neural network prediction model. Wang et al. [22] employed CNN to exploit higher-level features automatically, with N-words and entity mention features around candidate triggers. Rahul et al. [23] utilized bidirectional LSTM and GRU to identify triggers respectively. They extract the higher level features across the sentence. The previous work [24] have proposed a Bi-LSTM model integrating attention mechanism and sentence vector for biomedical event trigger detection. Chen [25] proposed a generalized cross-domain neural network transfer learning architecture and approach, which can share as much knowledge as possible between the source and target domains. More neural networks have focused on the sub-tasks of event extraction, such as event trigger identification [21–24], and relation classification [26–30]. Most of these deep models achieve superior performance compared to the traditional shallow methods.

It is worth mentioning that the biomedical event extraction task mainly includes two public datasets: MLEE corpus and BioNLP series corpora. The problem of data sparse is serious in BioNLP corpus. For example, in BioNLP 11 data set, the negative instances of trigger words in the training set account for 95% of the total number. Liu et al. [31] pointed out that data sparsity is an important factor affecting the performance of event extraction. In addition, when deep neural network model is used for classification, the context needs to be introduced to obtain the semantic information of the current word. A large number of irrelevant noise information will be introduced when the problem of data sparsity is serious, which may affect the performance of neural network. However, the statistical machine learning-based methods don’t need to learn contextual semantic information and features are relatively accurate, so corpus distribution has little significant impact on the performance. Therefore, most biomedical event extraction approaches (including the proposed method) based on neural network employ MLEE corpus as the benchmark dataset, such as references [17, 21–25], and some statistical machine learning-based methods also employ MLEE corpus, such as [10–13].

Although the above approaches have their notable advantages, certain challenges still remain: (1) The argument structures of simple events and complex events are different. In simple events, the arguments are only the relation pairs of (trigger, entity). However, arguments in the complex events may also be relation pairs of (trigger, trigger). However, existing biomedical event extraction methods usually deal with simple and complex events uniformly, and the performance of complex event extraction is low. (2) The interaction among arguments is not considered, which can improve the performance of complex event extraction. (3) Sentence level information is rarely exploited, which is helpful for detecting some ambiguous event types.

In light of these challenges, we propose a fine-grained biomedical event extraction method integrating sentence embeddings and multi-level attention mechanism. The main contributions are summarized as follows: (1) To improve the performance of complex biomedical event extraction, we design a fine-grained model which deal with simple and complex events respectively. (2) We propose a multi-level attention to enhance the interactions among the relevant arguments, which can further improve the performance of complex event extraction. (3) Sentence embeddings are integrated to exploit global sentence information, which is beneficial to detect some ambiguous event types.

## Results

### Corpus and evaluation

**Fig. 2:**
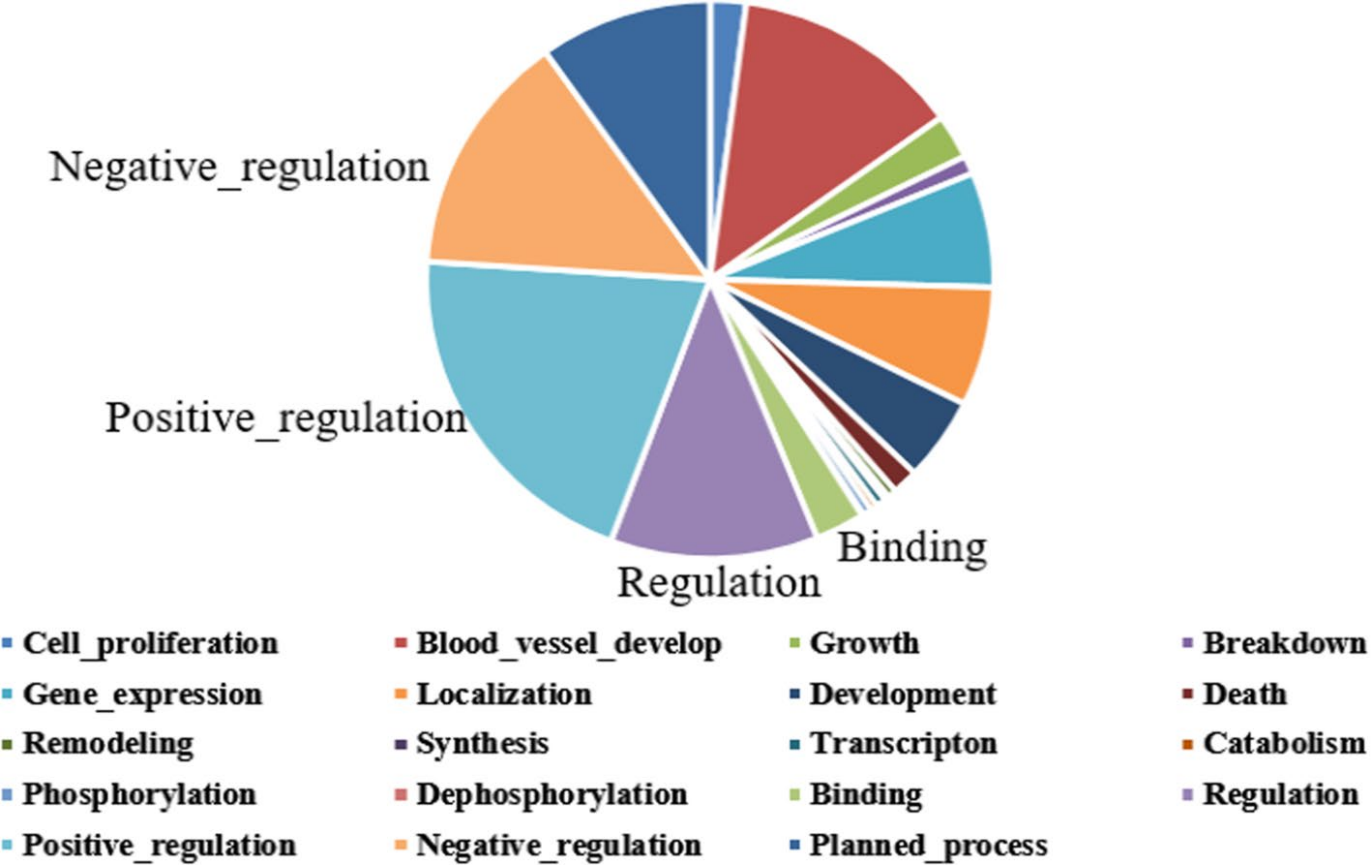
The distribution of the event types on the MLEE corpus

The commonly used dataset (MLEE) [10] is employed in our experiments. The MLEE corpus covers from the molecular level to the whole organism biomedical organizations. Table 1 illustrate the static distribution of the MLEE dataset. From Table 1, there are 262 event documents, 2608 sentences and 6677 events in total. The biomedical event types are divided into four categories, including Anatomical, Molecular, General and Planned, which can be further divided into 19 sub-classes. As shown in Fig. 2, the four types of complex biomedical events (*Regulation*, *Positive_regulation*, *Negative_regulation*, *Binding*) occupy a large proportion in the corpus. Therefore, the complex biomedical event extraction is important to improve the overall the performance of biomedical event extraction.

**Table 1:** The static distribution of MLEE corpus

Data	Train	Validation	Test	Total
Documents	206	30	59	295
Sentences	1825	260	523	2608
Events	4673	668	1336	6677

We employ the evaluation criteria with P(recision)/R(ecall)/F(-score). The evaluation metric P/R/F is defined as below (1), where *TP, FP* and *FN* are short for True Positives, False Positives and False Negatives respectively.

1 $$P = \, \frac{TP}{{TP + FP}}{, }R = \frac{TP}{{TP + FN}}{, }F - score = \frac{2*P*R}{{P + R}}$$

### Hyper-parameters

We combine the train and validation datasets for training, use validation dataset for tuning parameters, and select the average parameters. The size of the word embeddings and sentence embeddings is 200. The number of Bi-LSTM neural network layer is 2, the batch_size is set to 64. The dropout rate is set to 0.5 for avoiding the overfitting. The number of hidden nodes is set to 200, the number of iterations is set to 100. We employ Adadelta as the stochastic-gradient descent algorithm. The learning rate is selected as 0.001 from the set {0.01, 0.001, 0.0001}.

### Experimental results of trigger identification

#### The effectiveness of sentence embeddings

To verify the efficiency of the sentence embeddings established to enrich the global sentence information, we design a baseline model for comparison (Table 2, line 1), which is based on the Bi-LSTM with dependency-based word embddings. We calculate the average and sum value of pre-trained word embeddings only, fine-tuned word embeddings only, the difference or summation between the pre-training word embddings and fine-tuning word embddings respectively. Finally, averaging the difference between the pre-trained word embeddings and fine-tuned word embeddings obtains the best performance. As shown in Table 2 (line 2), the F-score has been increased to 77.96%, improved by 3.75% significantly. This indicates the benefit of sentence embeddings for biomedical event trigger identification.

**Table 2:** Performance of different trigger identification models

Model	Precision (%)	Recall (%)	F-score (%)
Bi-LSTM	76.26 ± 0.52	72.27 ± 0.41	74.21 ± 0.40
Bi-LSTM + SE	82.81 ± 0.34	73.66 ± 0.39	77.96 ± 0.36
Bi-LSTM + Att	81.47 ± 0.41	75.55 ± 0.38	78.40 ± 0.39
Bi-LSTM + SE + Att	82.01 ± 0.27	78.02 ± 0.29	79.96 ± 0.27

*SE* Sentence Embeddings, *Att* Attention

#### The effectiveness of word level attention

The word level attention can filter out the irrelevant noise information and enhance the important words in the sentence. As shown in Table 2 (line 3), after integrating word level attention based on the baseline model, the F-score achieves 78.40%. Furthermore, when we integrate both sentence embeddings and word level attention, the model obtains the best performance, achieving 79.96% F-score. This indicates the word level attention can contribute to the task.

### Experimental results of event extraction

#### The effectiveness of multi level attention

To verify the efficiency of multi level attention, we build three different models as shown in Table 3: Bi-LSTM + Word level attention (line 2), Bi-LSTM + Sentence level attention (line 3), and Bi-LSTM + Multi level attention (line 4). As shown in Table 3, the F-scores of biomedical event extraction with word level attention and sentence level attention are both improved than the baseline Bi-LSTM model (line 1). However, when the multi level attention is integrated, the performance of biomedical event extraction is best, achieving 59.61%. This indicates the effectiveness of the multi level attention.

**Table 3:** The effectiveness of multi level attention for event extraction

Model	Precision (%)	Recall (%)	F-score (%)
Bi-LSTM	90.93 ± 0.35	38.50 ± 0.41	54.09 ± 0.37
Bi-LSTM + WAtt	90.75 ± 0.22	43.00 ± 0.25	58.35 ± 0.23
Bi-LSTM + SAtt	89.69 ± 0.23	44.12 ± 0.28	59.14 ± 0.27
Bi-LSTM + MultiAtt	90.24 ± 0.19	44.50 ± 0.16	59.61 ± 0.18
Bi-LSTM + MultiAtt + Fine-grained	91.05 ± 0.27	44.68 ± 0.31	59.94 ± 0.29

*WAtt* Word level attention, *SAtt* Sentence level attention, *MultiAtt* Multi level attention

To further verify the effectiveness of the multi level attention for complex biomedical event extraction, we list the F-scores of 19 biomedical event subclasses integrating word level attention and multi level attention respectively in Table 4. It can be found that, after adding multi level attention, the F-scores of complex biomedical events have been improved significantly than integrating word level attention only. In addition, among the 15 simple event types, the F-scores of 6 types of event extraction with multi level attention are higher than that of the word level attention model only; the F-scores of 6 types of event extraction with multi level attention is the same as or almost equal to that of the word level attention model. Only in *Transcription* and *Phosphorylation* types, the word level attention model achieves better performance. However, the two types only account for 0.56% and 0.51% of the total number of events. As Table 4 shown, the performance of simple event extraction is not significantly improved by multilevel attention. It may be because that simple events are composed of one trigger word and one argument, while complex events contain multiple arguments. The sentence level attention mechanism is used to enhance the interaction among multiple relevant arguments with the same trigger word. Therefore, the impact on argument detection of simple events is limited.

**Table 4:** The effectiveness of multi level attention for sub classes

	Event type	F-score (%)	
		Word Att	Multi Att
Complex events	Binding	65.26	73.27
	Regulation	39.47	41.29
	Positive_regulation	41.14	43.97
	Negative_regulation	37.22	38.90
Simple events	Cell_proliferation	65.67	67.65
	Development	74.85	77.71
	Blood_vessel_develop	97.31	95.73
	Growth	33.33	33.33
	Death	53.85	56.60
	Breakdown	64.71	70.27
	Remodeling	66.67	66.67
	Synthesis	0.00	0.00
	Gene_expression	69.67	69.14
	Transcription	76.19	47.06
	Catabolism	33.33	33.33
	Phosphorylation	85.71	67.67
	Dephosphorylation	0.00	0.00
	Localization	53.48	57.87
	Planned_process	47.92	52.12

In conclusion, the multi level attention can improve the performance of most types of biomedical events extraction, especially for complex biomedical events extraction.

#### The effectiveness of fine-grained argument detection

According to the difference of argument structure between simple and complex biomedical events, we propose the fine-grained argument detection method. As shown in Table 3 (line 5), the F-score is improved by 0.33%, achieving 59.94%, also the precision and recall are improved. To verify the significance of the fine-grained argument detection model, we conduct a T-test on the results of 10 experiments, and t < 0.05, which means the improvement by fine-grained detection is significant. This indicates that the fine-grained argument detection is beneficial for biomedical event extraction.

### Comparisons with other methods

In this section, we list and compare the experimental results of biomedical trigger identification and event extraction with other advanced methods on the commonly used dataset MLEE.

#### Performance comparisons of trigger identification with other methods

As mentioned in the Related Work, there are some advanced approaches to detect event triggers. They are listed as follows.

- SVM1: a SVM based model proposed by Pyysalo et al. [10], which extracted rich hand-crafted features.
- SVM2: a semi supervised SVM based frame integrating hidden topics and hand-crafted features, which is proposed by Zhou et al. [11].
- EANNP: a neural network prediction model proposed by Nie [21], which introduced word embedding.
- CNN: a CNN-based classifier integrating multiple distributed representation, which is proposed by Wang et al. [17].
- GRU: a GRU neural network built by Rahul et al. [23], which introduced word and entity type embeddings.
- LSTM: A LSTM-based model integrating dependency word embeddings and word level attention, which is proposed in our previous work [24].
- LSTM + CRF: a LSTM + CRF model proposed by Chen [25], which integrated transfer learning architecture for trigger recognition.

Two-stage Method: A two-stage model proposed in the previous work [13], which is based on traditional machine learning methods.

Table 5 shows the comparison results of methods above, and we can find that:

1. The performances of EANNP, CNN, LSTM, GRU, LSTM + CRF and our proposed method are better than SVM classifiers on average F-score. It reveals the effectiveness of deep learning methods, which can obtain high semantic representations without artificial features.
2. The LSTM and GRU models achieve better performance than CNN model, which may verify the sequential model are more suitable for biomedical event extraction. Since there are usually many long texts in biomedical literature, and the recurrent neural network (LSTM and GRU) can capture global contextual information.
3. Our proposed model outperforms tthe state-of-the-art two-stage method [13]. Our previous two-stage method [13] is based on SVM classifier and PA algorithm, which divided the trigger identification into trigger recognition and trigger classification stages, and need to extract task-based hand-crafted features for each stage. The proposed model only need once classification, and the neural network can skip the step of extracting complex hand-designed features. The results illustrate the effectiveness of our biomedical event trigger identification method.

**Table 5:** Performance comparisons of trigger identification

Methods	Precision (%)	Recall (%)	F-score (%)
SVM1 [10]	70.79	81.69	75.84
SVM2 [11]	72.17	82.26	76.89
EANNP [21]	71.04	84.60	77.23
CNN [17]	80.60	74.23	77.82
GRU [23]	79.78	78.45	79.11
LSTM [24]	81.79	77.76	79.73
LSTM + CRF [25]	81.76	77.71	79.68
Two-stage[13]	79.16	80.35	79.75
**Ours**	82.01	78.02	79.96

#### Performance comparisons of event extraction with other methods

Due to the complexity of biomedical event extraction, the research on event extraction is less than that on trigger identification. Pyysalo et al. [10] proposed a SVM-based approach with rich hand-crafted features. It has significant potential over existing systems, and we select this method as the baseline method. Zhou et al. [11] proposed semi-supervised learning model for biomedical event extraction, which integrated hidden topics embedded in the sentences for describing the distance. Wang et al. [17] employed CNN for biomedical event extraction, which integrated multiple distributed features. The multiple distributed features contain word embeddings, trigger types, POS and topic representation. As shown in Table 6, our proposed method achieves an F-score of 59.94%, which is 1.63% higher than Wang et al.’s [17] CNN methods. The experimental results demonstrate the effectiveness of our proposed method.

**Table 6:** Performance comparisons of event extraction

Methods	Precision(%)	Recall (%)	F-score(%)
Pyysalo et al. [10]	62.28	49.56	55.20
Zhou et al. [11]	55.76	59.16	57.41
Wang et al. [17]	60.56	56.23	58.31
**Ours**	91.05	44.68	59.94

## Discussion

Experimental results show that the proposed biomedical event extraction method based on fine-grained and multi-level attention has good performance. The detailed analysis for the improvement is as follows:

### Sentence embeddings

Sentence embeddings can build the connection among different words and enrich the sentence level information. A sentence usually contains multiple events, which are related to each other. Moreover, there is usually a strong correlation between triggers and arguments, which is beneficial to the recognition of each. The semantic information of triggers or arguments is helpful to resolve the ambiguities for some types. For example, in the sentence “We especially focused on the role of Crk adaptor protein in EphB mediated signaling.”, the correct type of the event triggered by “mediated” is Positive_regulation. However, it might be easily misidentified as a Regulation trigger because in training set it also sometimes appears as a trigger of Regulation event. In this case, the global sentence level features are important. According to the other word “role” which always exists in Positive_regulation type event, and the word “signaling” which serves as an argument of “mediated”, it is more helpful to classify “mediated” correctly. Therefore, we construct the sentence embeddings to enrich global sentence information. The experimental results show that the sentence embeddings have improve the performance of biomedical event detection significantly.

### Fine-grained argument detection

In the simple events, there exists only one argument consisting of a trigger word and an entity. In the complex events, there are multiple arguments which consist of trigger word and entity or trigger word and trigger word (nested events). According to the different argument structures of simple and complex events, we propose a fine-grained argument detection model. Firstly, we construct different argument candidates for simple and complex events respectively. Then, the same argument types of simple and complex events are labeled, trained and classify separately. Thus the additional relationship between trigger and trigger in nested events is not easy to lose. For example, in the sentence of Fig. 1, besides the arguments of (over-expression, Bmi-1) and (promote, tumorigenesis), the argument relationship of (promote, over-expression) is more easily to identify by the fine-grained argument detection. Therefore, the performance of complex biomedical event argument detection is improved. The experimental results verify the effectiveness of the fine-grained argument detection model.

### Multi-level attention

Word level attention focuses on important words within one sentence, and sentence level attention enhances the interaction among sentences. In this work, we define the arguments with same trigger as relevant arguments, and integrate the multi-level attention to enhance the effect among the relevant arguments. The multi-level attention is helpful to identify each other among the relevant arguments. Taking the sentence in Fig. 4 as an example, the type of argument relationship (binding, TRAF2) is Theme. Considering the influence of relevant arguments, it is more easily to correctly judge the type of the argument relationship (binding, CD40) as Theme type. As shown in Table 4, the multi-level attention mechanism improves the performance of complex biomedical event extraction significantly, which proves the effectiveness of muliti-level attention.

## Methods

**Fig. 3:**
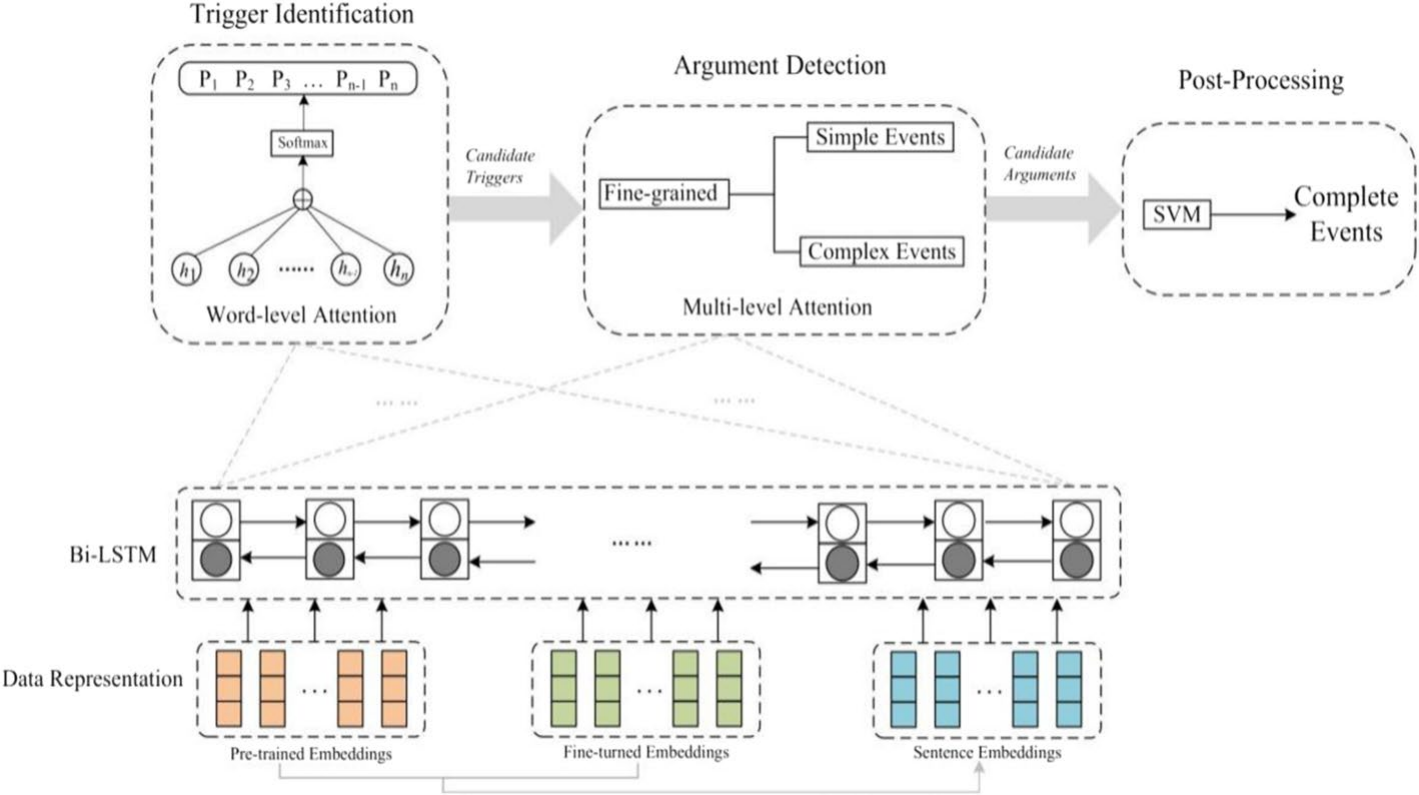
The overall architecture of biomedical event extraction

In this paper, we propose a fine-grained biomedical event extraction method based on sentence embeddings and multi-level attention mechanism. Figure 3 illustrates the structures of our model, which mainly contains five parts: (1) Data representation, which combines dependency-based word embeddings and sentence embeddings as input representation. (2) Bi-LSTM integrating reading gate, which is the basis neural network for trigger identification and argument detection. (3) Trigger identification, which divides each event trigger candidate to a concrete event type integrating word level attention. (4) Argument detection, which classifies each event argument candidate to a specific event argument type based on fine-grained detection and multi-level attention. (5) Post-processing, the complete biomedical events are generated by the post-processing.

### Data representation

#### Dependency-based word embeddings

Different from other NLP tasks, biomedical event extraction needs more information in dependency contexts than in linear contexts [32]. Therefore, we employ Word2vecf [33] to train dependency-based word embeddings as feature representation, which can capture more functional and less topical similarity, yielding more focused embeddings.

In this work, we download about 6G PubMed abstracts (from 2013 to 2019), and parse them with Gdep parser, which is a dependency parse tool specialized for biomedical texts. Then, we derive word contexts in syntactic relations and generate dependency based word embeddings by Word2vecf.

#### Sentence embeddings

The global information of the sentence is critical to biomedical event extraction. The previous work [24] has demonstrated the effectiveness of sentence embeddings for biomedical event extraction. With similar approach, two different kinds of word embeddings in the whole training process are employed. As (2) shown, *x*_*t*_ is the pre-trained dependency-based word embeddings, which can capture the potential feature information from large scale unlabeled corpus. *x*_*t*_’ is the fine-tuned word embeddings which contain rich information associated with the biomedical events. The initial value of xt’ is the same as that of pre-trained word embedding xt, and then it will be updated with the neural network training. The sentence vector *d*_*0*_ is obtained from the average value of the difference between the two aforementioned embeddings of all the word in the sentence, *n* is the length of the sentence, *t* refers to the current time, *T* denotes the total training time, and *n* is the length of the sentence. To control what information should be retrained for future time steps, we add a reading gate $r_{t} \in \left[ {0,1} \right]^{n}$ based on the original Bi-LSTM neural network.

2 $$d_{0} = \frac{1}{n}(\sum\limits_{t = 1}^{T} ( x^{\prime}_{t} - x_{t} ))$$

### Bi-LSTM integrating reading gate

Bi-LSTM includes the forward LSTM and backward LSTM to better learn the context representation from the two directions. As (3) shown, the forward pass output ($h_{t}^{b}$) and the backward pass output ($h_{t}^{f}$) are combined by summation.

Our new Bi-LSTM architecture leveraged by both dependency-based word embeddings *x*_*t*_ and fine-tuned word embeddings *x*_*t*_’ is described as (4) to (7). A standard architecture of LSTM mainly consists of three units, which are the input, output, and forget gates respectively. As (8) shown, a reading gate is added to control the sentence embeddings. (9) describes the sentence information at *t* moment. The cell value $c_{t}$ is modified to (10) after integrating sentence embeddings.

3 $$h_{t} = [\mathop {h_{t} }\limits^{ \to } \oplus \mathop {h_{t} }\limits^{ \leftarrow } ]$$

4 $$i_{t} = \sigma (x_{t} \cdot w_{xh}^{i} + x^{\prime}_{t} \cdot w_{{x^{\prime}h}}^{i} + h_{t - 1} \cdot w_{{hh^{\prime}}}^{i} + b_{h}^{i} )$$

5 $$f_{t} = \sigma (x_{t} \cdot w_{xh}^{f} + x^{\prime}_{t} \cdot w_{{x^{\prime}h}}^{f} + h_{t - 1} \cdot w_{{hh^{\prime}}}^{f} + b_{h}^{f} )$$

6 $$o_{t} = \sigma (x_{t} \cdot w_{xh}^{o} + x^{\prime}_{t} \cdot w_{{x^{\prime}h}}^{o} + h_{t - 1} \cdot w_{{hh^{\prime}}}^{o} + b_{h}^{o} )$$

7 $$\tilde{c}_{t} = \tanh (x_{t} \cdot w_{xh}^{c} + x^{\prime}_{t} \cdot w_{{x^{\prime}h}}^{c} + h_{t - 1} \cdot w_{{hh^{\prime}}}^{c} + b_{h}^{c} )$$

8 $$r_{t} = \sigma (x_{t} \cdot w_{xh}^{r} + x_{t}^{^{\prime}} \cdot w_{x^{\prime}h}^{r} + h_{t - 1} \cdot w_{hh^{\prime}}^{r} + b_{h}^{r} )$$

9 $$d_{t} = r_{t} \odot d_{t - 1}$$

10 $$c_{t} = i_{t} \odot \tilde{c}_{t} + f_{t} \odot c_{t - 1} + \tanh (d_{t} )$$

where *x* is the input embeddings at *t* moment. *i*, *f*, *o* and *c* are input gate, forget gate, output gate and the proposed values respectively. $w_{xh}$ is the input connections, $w_{hh}$ is recurrent connections, and *b*_*h*_ is the bias value. *σ* represents the logistic sigmoid function, ⊙ denotes the element-wise multiplication, and *c*_*t*_ means the true cell value at time *t*.

### Trigger identification

Trigger identification aims to assign each token or phrase to a specific event trigger type or a negative class if it does not belong to any trigger class. It is usually treated as a multi-classification problem. In this paper, we mark each candidate trigger in a given sentence by BIO labeling method [34]. Then we build a Bi-LSTM trigger identification model, and integrate word level attention to enhance the important word information in the sentence.

#### Word level attention

According to the analysis of corpus, different words in a sentence usually have different influence in the overall semantic information. Therefore, we integrate word level attention to filter out the irrelevant noise information and enhance the important words. Firstly, we initialize a random weight matrix tuned with the training process. Then, the weight vector could learn word features automatically and record the significant information by increasing the corresponding weights.

As shown in (11), we employ the activation function tanh to handle the final state *H*($H \in R^{{d_{w} \times L}}$),where *L* is the sentence length, *d*_*w*_ denotes the word embeddings dimension.

11 $$N = \tanh (H)$$

In (12), the attention mechanism will produce a vector *α* of attention weights, where *w* refers to a trained parameter vector and *w*^*T*^ is the transpose of *w*. Then, in (13), a weighted representation *γ* is formed by a weighted sum of the output vectors *H*. At last, the overall semantic information of the sentence is obtained from (14), where $h_{i}^{*}$ represents the final sentence representation. The dimension of *α*, $w$, *γ* and $h_{{}}^{*}$ is *L*, *d*_*w*_, *d*_*w*_, *d*_*w*_ separately.

12 $$\alpha = soft\max (w^{T} N)$$

13 $$\gamma = H\alpha^{T}$$

14 $$h^{*} = \tanh (\gamma )$$

#### Trigger classification

In this work, we treat each token of sentences as a trigger candidate instance. Trained by the Bi-LSTM model based on attention mechanism, the hidden output $h_{i}^{*}$ of each word is generated. Then, we utilize softmax function as classifier to predict label $\hat{y}$ of each trigger candidate. The classifier takes the hidden output $h_{i}^{*}$ as input:

15 $$\hat{p}(y|x) = soft\max (Wh_{i}^{*} + b)$$

16 $$\hat{y} = \arg \mathop {\max }\limits_{y} \hat{p}(y|x)$$

In our model, the objective function is the cross-entropy loss defined as (16). In (16), $t_{i}^{j}$ denotes the *j*-th type distribution of the *i*-th instance, and $\hat{p}_{i}^{j}$ is the predicted distribution.

17 $$L(\theta ) = - \sum\limits_{i}^{{}} {\sum\limits_{j}^{{}} {t_{i}^{j} \log (\hat{p}_{i}^{j} )} }$$

### Argument detection

Argument detection belongs to complex relation classification. In the simple events, argument detection aims to find the relation between predicted trigger and entities in sentence. In the complex events, it aims to find the relation between predicted trigger and entities or other triggers (nested event). Then, if the relation exists, the relation types should be given.

#### Fine-grained argument detection

Considering the differences of argument structure between simple biomedical events and complex events, we propose the fine-grained argument detection method to further improve the performance of complex biomedical event extraction.

**Fig. 4:**
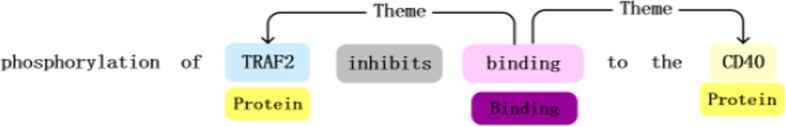
An example of “Binding” type biomedical event

1. We construct different argument candidates for simple and complex events respectively. For simple events, we take the sentence fragments composed of predicted trigger, entity and other words between them as argument candidate instances. For complex events, the argument candidate instances are composed of predicted trigger, entity/trigger and other middle words.
2. We make a fine-grained distinction between the same type arguments in simple and complex events. For example, we lable the *Theme* type arguments in simple events as “Theme”, lable the same type arguments in complex events as “CTheme”, then train and classify them separately.
3. According to the analysis of arguments structure in complex events, we find that the argument relation pairs in complex events have the same trigger, and these arguments usually have strong interaction. For example, as Fig. 4 shown, the argument relation (binding, TRAF3) and (binding, CD40) have the same trigger “binding”, also they belongs to the same type *Theme*, and they are in the same complex event. In addition, the arguments with same trigger in simple events also have common features. Therefore, we define these arguments as relevant arguments, and employ multi level attention to enhance their interaction.

**Relevant arguments** Arguments containing the same trigger word in biomedical events.

#### Multi level attention

Word level attention can obtain the key semantic information within a given sentence. Sentence level attention introduces global semantic information, and enhances the interaction among relevant arguments. To take the above advantages, we propose a multi level (word level and sentence level) attention for argument detection.

In this work, the relevant argument instances are represented as vector matrix $H^{*} = \{ h_{1}^{*} ,h_{2}^{*} , \cdots ,h_{M}^{*} \}$, where $h_{i}^{*}$ is the hidden output of the word level attention layer, *M* is the number of relevant instances within the same batch. As shown in (18), after reducing the dimension of $h_{i}^{*}$, a new vector matrix $H_{S}^{*} = \{ h_{{S_{1} }}^{*} ,h_{{S_{2} }}^{*} , \cdots ,h_{{S_{M} }}^{*} \}$ representing the sentence feature is generated. As shown in (19)–(22), the weighted hidden output by the sentence attention is obtained, and it will be sent to softmax function for argument prediction.

18 $$h_{{S_{i} }}^{*} = \sum\limits_{i = 1}^{{d_{w} }} {\sum\limits_{j = 1}^{L} {h_{i}^{*} /L} }$$

19 $$N{ = }\tanh (H_{S}^{*} )$$

20 $$\alpha { = }soft\max (w^{T} N)$$

21 $$\gamma_{i} = h_{{S_{i} }} \alpha_{i}$$

22 $$h_{{S_{i} }}^{*} = \tanh (\gamma_{i} )$$

#### Argument prediction

To improve the performance of argument detection, the same argument types in simple and complex biomedical events are divided into more fine-grained categories, labeled and classification respectively. After the Bi-LSTM and multi level attention layer, the hidden output of Eq. (22) is sent to softmax function to get the argument candidate type, as shown in (23) and (24).

23 $$\hat{p}\left( {y_{{S_{i} }} \left| S \right.} \right) = soft\max \left( {Wh_{{S_{i} }}^{*} + b} \right)$$

24 $$\mathop {y_{{S_{i} }} }\limits^{ \wedge } = \arg \mathop {\max }\limits_{y \in C} \hat{p}\left( {y_{{s_{i} }} |S} \right)$$

where *W* is the learning matrix, *b* is the bias value, and *C* denotes the set of argument types. The objective function is the cross-entropy loss function.

### Post-processing

Pipeline biomedical event extraction methods include three sub processes: trigger identification, argument detection, and post-processing. The post-processing can remove invalid event candidates and ensure the final events correctly [35]. In this paper, we utilize SVM classifier based on TEES [15] to learn the legal event structure automatically by the extracted features, and then constitute correct event candidates. The features extracted in this process mainly include three categories [36]: linear span features, such as bag-of-words between arguments; argument combination features, such as argument role features and count features; argument content features, such as entity features and argument edge features.

## Conclusions

In this paper, we present a novel fine-grained biomedical event extraction method based on sentence embeddings and multi-level attention. The sentence embeddings enrich the global sentence-level information and obtain abundant contextual information related to events within a sentence. The fine-grained argument detection model deals with the simple and complex biomedical events respectively, which can improve the performance of complex biomedical event extraction. Furthermore, we enhance the interactions among relevant arguments and obtain the most important information by the multi-level attention mechanism. Experimental results conducted on a real-word multi-level event extraction (MLEE) corpus dataset demonstrate the effectiveness of our proposed method.

## Data Availability

The datasets that used in experiments are available online at http://nactem.ac.uk/MLEE.

## References

[1] Chung-Chi H, Lu Z (2016). Community challenges in biomedical text mining over 10 years: success, failure and the future. Brief Bioinform.

[2] Sophia T, Paul NR (2015). Event-based text mining for biology and functional genomics. Brief Funct Genomics.

[3] Ohta T, Pyysalo S, Rak R, et al. Overview of the pathway curation (pc) task of bionlp shared task 2013. In: Proceedings of the BioNLP shared task 2013 workshop, 2013. p. 67–75.

[4] Kim JD, Ohta T, Pyysalo S, et al. Overview of BioNLP'09 shared task on event extraction. In: The workshop on current trends in biomedical natural language processing: shared task, Boulder, Colorado, 2009. p. 1–9.

[5] Kim JD, Wang Y, Takagi T, et al. Overview of genia event task in BioNLP shared task 2011. In: Bionlp shared task 2011 workshop, Portland, Oregon, USA, 2012. p. 7–15.

[6] Kim JD, Wang Y, Yasunori Y, et al. The genia event extraction shared task, 2013 edition-overview. In: Bionlp shared task 2013 workshop, Sofia, Bulgaria, 2013. p. 8–15.

[7] Deléger L, Bossy R, Chaix E, et al. Overview of the bacteria biotope task at BioNLP shared task 2016. In: Bionlp shared task workshop-association for computational linguistics, Berlin, Germany, 2017. p. 12–22.

[8] Móra G, Farkas R, Szarvas G, et al. Exploring ways beyond the simple supervised learning approach for biological event extraction. In: Proceedings of the BioNLP 2009 workshop companion volume for shared task, 2009. p. 137–40.

[9] Bui QC, Campos D, van Mulligen E, et al. A fast rule-based approach for biomedical event extraction. In: Proceedings of the BioNLP shared task 2013 workshop, 2013. p. 104–8.

[10] Pyysalo S, Ohta T, Miwa M (2012). Event extraction across multiple levels of biological organization. Bioinformatics.

[11] Zhou D, Zhong D (2015). A semi-supervised learning framework for biomedical event extraction based on hidden topics. Artif Intell Med.

[12] Zhou D, Zhong D, He Y (2014). Event trigger identification for biomedical events extraction using domain knowledge. Bioinformatics.

[13] He X, Li L, Liu Y, Xiaoming Y, Meng J (2018). A two-stage biomedical event trigger detection method integrating feature selection and word embeddings. IEEE/ACM Trans Comput Biol Bioinform.

[14] Björne J, Heimonen J, Ginter F (2011). Extracting contextualized complex biological events with rich graph-based feature sets. Comput Intell.

[15] Björne J, Salakoski T (2015). TEES 2.2: biomedical event extraction for diverse corpora. BMC Bioinform.

[16] Miwa M, Ananiadou S (2015). Adaptable, high recall, event extraction system with minimal configuration. BMC Bioinform.

[17] Wang A, Wang J, Lin H (2017). A multiple distributed representation method based on neural network for biomedical event extraction. BMC Med Informa Decis Mak.

[18] Li L, Wan J, Zheng J (2018). Biomedical event extraction based on GRU integrating attention mechanism. BMC Bioinforma.

[19] Yan S, Wong K (2019). Context awareness and embedding for biomedical event extraction. Bioinformatics.

[20] Abdulkadhar S, Bhasuran B, Natarajan J (2020). Multiscale Laplacian graph kernel combined with lexico-syntactic patterns for biomedical event extraction from literature. Knowl Inf Sys.

[21] Nie Y, Rong W, Zhang Y (2015). Embedding assisted prediction architecture for event trigger identification. J Bioinform Comput Biol.

[22] Wang J, Zhang J, Yuan A, et al. Biomedical event trigger detection by dependency-based word embedding. In: IEEE international conference on bioinformatics and biomedicine. p. 429–32. 10.1186/s12920-016-0203-8 PMC4980775 27510445

[23] Rahul PV, Sahu SK, Anand A. Biomedical event trigger identification using bidirectional recurrent neural network based models. arXiv: Computation and Language, 2017. p. 316–21.

[24] He X, Li L, Wan J, et al. Biomedical event trigger detection based on BiLSTM integrating attention mechanism and sentence vector. In: IEEE international conference on bioinformatics and biomedicine (BIBM), 2018. p. 651–4.

[25] Chen Y (2019). Multiple-level biomedical event trigger recognition with transfer learning. BMC Bioinform.

[26] Raj D, Sahu S, Anand A. Learning local and global contexts using a convolutional recurrent network model for relation classification in biomedical text. In: Proceedings of the 21st conference on computational natural language learning (CoNLL 2017). 2017. 10.18653/v1/k17-1032.

[27] Zheng S, Hao Y, Lu D (2017). Joint entity and relation extraction based on a hybrid neural network. Neurocomputing.

[28] Li F, Zhang M, Fu G (2017). A neural joint model for entity and relation extraction from biomedical text. BMC Bioinforma.

[29] Miwa M, Bansal M. End-to-end relation extraction using LSTMs on sequences and tree structures. In: Proceedings of the 54th annual meeting of the association for computational linguistics. 2016. 10.18653/v1/p16-1105.

[30] Zhao A, Qi L, Dong J, Yu H (2018). Dual channel LSTM based multi-feature extraction in gait for diagnosis of neurodegenerative diseases. Knowl-Based Syst.

[31] Liu S, Chen Y, Liu KK, et al. Exploiting argument information to improve event detection via supervised attention mechanisms. In: Proceedings of the 55th Annual Meeting of the Association for Computational Linguistics, 2017. p. 1789–98.

[32] Miwa M, Pyysalo S, Hara T, et al. Evaluating dependency representation for event extraction. In: International conference on computational linguistics, 2010. p. 779–87.

[33] Levy O, Goldberg Y. Dependency-based word embedding. In: Meeting of the association for computational linguistics. 2014, 302–8.

[34] Gupta P, Schütze H, Andrassy B. Table filling multi-task recurrent neural network for joint entity and relation extraction. In: Proceedings of COLING 2016, the 26th international conference on computational linguistics, Osaka, Japan, 2016. p. 2537–47.

[35] Björne J (2009). Extracting complex biological events with rich-based feature sets. Computational Intelligence.

[36] Björne J (2014). Biomedical event extraction with machine learning. TUCS Diss.

